# A toolbox for navigating and analyzing the spatiotemporal properties of retinal waves

**DOI:** 10.1101/2025.10.23.684204

**Published:** 2025-10-24

**Authors:** Kaylee M. Yeager, Rishikesh K. Gupta, Elysia A. Gauthier, Alexander J. Fisch, Natalie A. Clark, Fernanda S. Orsi, Alexandre Tiriac

**Affiliations:** 1. Department of Biological Sciences, Vanderbilt University, Nashville, TN 37232, USA; 2. Department of Pharmacology, Vanderbilt University, Nasvhille, TN 37232, USA; 3. Vanderbilt Brain Institute, Vanderbilt University, Nashville, TN 37232, USA; 4. Department of Ophthalmology and Vision Sciences, Vanderbilt University, Nashville, TN 37232, USA

## Abstract

The precise development of the visual system is driven by retinal waves, which are bursts of spontaneous activity that propagate across retinal neurons in a wave-like fashion. In mice, retinal waves begin embryonically and continue until eye opening at the end of the second postnatal week. During this time, the mechanisms for generating and propagating retinal waves are changing, thus causing retinal waves to exhibit highly dynamic spatiotemporal properties from one day to the next. Critically, the spatiotemporal properties of retinal waves have been shown to instruct the development of the visual system, including eye-specific segregation, retinotopic mapping, direction selectivity, and potentially retinal vascularization. Currently, there is no method for the automatic detection and high-throughput analysis of the spatiotemporal properties of retinal waves. To overcome this barrier, we have created a toolbox to automatically detect retinal waves and analyze their spatiotemporal properties from data collected using multielectrode arrays or calcium imaging. We apply this toolbox to enrich our understanding of retinal waves. First, we recapitulate and uncover novel dynamic spatiotemporal properties of retinal waves in the first two postnatal weeks. Second, we apply this toolbox on ultra long physiological recordings to demonstrate that the spatiotemporal properties of waves change throughout the day in an age-dependent manner. Third, we demonstrate that this toolbox can detect waves in the presence of pharmacological agents that increase the baseline firing of neurons, potentially enabling the discovery of novel factors that perturb retinal waves and visual development. Our toolbox is an intuitive interface that provides a systemized method to segregate and analyze retinal waves and other wave-like data.

## One Sentence Summary

We present a toolbox for the automatic detection and analysis of retinal waves, which accurately captures their spatiotemporal dynamics across development and reveals new insights.

## Introduction

During early development of the vertebrate visual system and before the onset of vision, the retina generates waves of spontaneous activity that propagate across the retinal ganglion cell (RGC) layer^1–3^. These retinal waves are critical for refining topographic maps, eye-specific segregation, and the emergence of feature selectivity in visual targets downstream of the retina, such as the dorsal lateral geniculate nucleus (dLGN), superior colliculus (SC), and primary visual cortex^4–7^. In mice, retinal waves emerge in three distinct stages that correspond to the dynamic development of retinal circuits. Stage I waves, occurring from embryonic day 16 (E16) through birth, are mediated by gap junctions and nicotinic acetylcholine receptors (nAChRs), and display diffuse, spatially variable activation^8^. Stage II waves, dominant during the first postnatal week (P1–P10), are driven by cholinergic signaling among starburst amacrine cells via β2-containing nAChRs, and exhibit coherent, slowly propagating activity across large retinal territories^9,10^. Stage III waves, emerging around P10, are glutamatergic, initiated by bipolar cells, and propagate more rapidly, activating ON and OFF RGCs sequentially^11,12^.

Retinal waves are spontaneous but not random: they exhibit structured spatiotemporal properties^13^ that contribute meaningfully to circuit development. These include defined wave frequencies (approximately once per minute in Stage II), spatial extent, and speeds that increase with age. One striking feature is the emergence of directional propagation, particularly a temporal-to-nasal bias during a transient window (P9–P12), which mimics optic flow experienced during forward locomotion^14–16^. This directionality has been shown to prime the development of motion-sensitive circuits in the SC^14^. Other spatiotemporal properties also matter: the slow frequency of stage II waves increases the likelihood of asynchronous activity between the two eyes which is thought critical for the development of eye-specific segregation^17^ and the area of waves matters for retinotopic mapping in binocular regions of the SC^18^. Lastly, the very act of a propagating wave leads to a local synchrony of neurons that is critical for Hebbian plasticity refinement of retinal axons to the SC^19^. The development of retinal vasculature is also thought to depend on retinal waves^20,21^ but whether this is done in an instructive or permissive manner is unknown. Across all stages, the patterned activity of retinal waves reflects the organization of the underlying circuitry and provides essential instructive signals for synaptic refinement and visual map formation before the onset of sensory experience.

Despite the recognized importance of retinal waves and their spatiotemporal dynamics, there is currently no widely adopted toolbox for automatic detection and characterization of retinal waves. Existing approaches are typically modality-specific, often rely on manual curation, and are based on custom algorithms that are not easily accessible. These limitations pose challenges for studying how wave properties evolve over time, respond to perturbations, or vary across genetic backgrounds. Moreover, defining discrete wave events in noisy or complex datasets, whether from age-related or introduction of pharmacological agents, remains a technical hurdle. In this study, we introduce a new analytical toolbox designed to automatically segment and quantify retinal waves from data obtained using high-density multielectrode arrays and calcium imaging. Our method robustly captures known spatiotemporal features of waves across developmental stages. We further demonstrate its utility in long recordings and under conditions of neural perturbation, providing novel insights about retinal waves. We also created a user-friendly application that utilizes all the tools present in our toolbox, which facilitates navigation and quality control of datasets. By standardizing wave detection and enabling new kinds of analyses, this toolbox aims to promote reproducibility and accelerate discovery of novel factors that govern retinal waves and early visual system development.

## Results

### A toolbox to detect retinal waves and analyze their spatiotemporal properties

**Figure 1:**
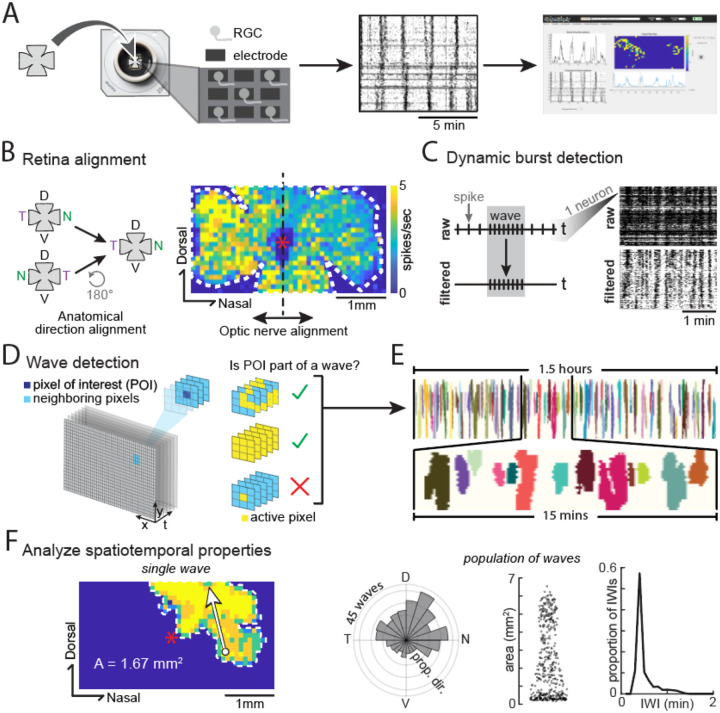
A toolbox to detect retinal waves and analyze their spatiotemporal properties. A) Schematic showing how a retina is mounted on a high-density multi-electrode array (HD-MEA) chip (left), a raster plot of the recorded activity in a P8 retina (middle), and a screenshot of the user interface (GUI) after the recorded data has been uploaded. B) Schematic of the toolbox’s retina alignment feature. Heatmap shows the average activity of each electrode over time in a recording of a P12 retina (same recording in C-F). C) Schematic showing how the toolbox’s burst detection analysis filters raw data. D) Schematic demonstrating how the toolbox detects and segregate retinal waves. E) Representative image of the retinal waves detected from a recording. Each color represents a different wave ID. F) Analysis of the spatiotemporal properties of a single wave (left) and the population of waves (right, N=435 waves). Abbreviations: RGC – retinal ganglion cells, D – dorsal, N – nasal, V – ventral, T – temporal, AP – action potential, IWI – inter-wave interval.

We developed a toolbox, called “WaveMiner”, capable of analyzing the comprehensive spatiotemporal dynamics of spontaneous retinal waves from *ex vivo* high-density multielectrode array (HD-MEA) recordings of retinal activity (Figure 1A). We developed the toolbox to either load data from the Maxwell HD-MEA system or to load virtually any retinal wave data that is structured in a way that contains both spatial and temporal information about neural activity (see methods). An initial module of the toolbox spatially aligns data across retinal wave experiments (temporal retina is always on the left, ventral retina is always on the bottom, and optic nerve is always at the center) by flipping the data and/or offsetting it, allowing for simple and accurate regional spatiotemporal comparisons across recordings (Figure 1B).

Before detecting the waves, the toolbox pre-processes the data by running a “dynamic burst detection” analysis to filter out tonic activity and keep phasic activity (which includes both propagating waves and transient, non-propagating events) (Figure 1C). The purpose of this phasic activity filtering is to allow the toolbox to detect waves in different conditions, especially ones where waves are present in conjunction with high tonic firing rates, like stage III waves or with pharmacological agents.

Once only phasic activity remains, the toolbox applies a 3D flood filling algorithm that automatically detects retinal waves. The algorithm is anchored on the definition that a unique wave constitutes any cluster of pixels connected in time (*t*) and space (*x* and *y*). To detect waves according to this definition, the algorithm scans every pixel of the xyt data matrix containing only phasic neural activity. Upon finding a pixel with phasic activity, the algorithm begins segregating a wave from that pixel of interest (POI) and analyzes neighboring pixels for phasic activity (Figure 1D). The process repeats until all active pixels connected in time and space are collected, forming a distinct retinal wave (more detailed information about this algorithm in methods section). Our algorithm successfully and reliably segregates retinal waves over hours of recordings (Figure 1E). Following wave detection, the toolbox enables analysis of the spatiotemporal properties of individual waves (Figure 1F, **left**) or populations of waves (Figure 1F, **right**).

### Validating our toolbox on known spatiotemporal properties of waves across development

**Figure 2:**
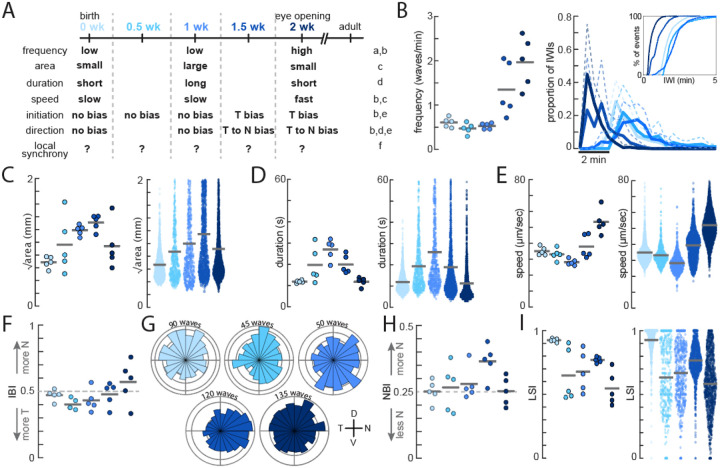
Using the toolbox to observe dynamic trends of the spatiotemporal properties of waves throughout development. A) Developmental timeline of the spatiotemporal properties of retinal waves in mice. Age groups are defined as 0 weeks (P0-P2, N=1182 waves across 5 mice), 0.5 weeks (P3-P5, N=575 waves across 5 mice), 1 week (P6-P8, N=691 waves across 5 mice), 1.5 weeks (P9-P11, N=1471 waves across 5 retinas), and 2 weeks (P12-P14,N=1950 waves across 5 retinas). References: a^24^, b^25^, c^13^, d^27^, e^29^, f^24^. B) Frequency (left) of waves per mouse across age groups. Inter-wave intervals across age groups, shown as a proportion of events at a given interval length and as a cumulative distribution function (right). C-E) Wave size (calculated as $\sqrt{\text{Area}}$), speed, and duration of waves across age groups per mouse (left) and per wave (right). F) Proportion of nasally initiated waves (IBI) per mouse across ages groups, analyzed by halves (temporal vs. nasal retina). G) Propagation direction of waves across ages groups. H) Proportion of waves that propagated in the nasal direction (NBI), analyzed by quadrants (temporal, nasal, ventral, and dorsal retina). I) Ratio of activity ahead of wavefront versus activity within wavefront (LSI) per mouse (left) and per wave (right). Abbreviations: wk – week, IWI – inter-wave interval, IBI – initiation bias index, D – dorsal, N – nasal, V – ventral, T – temporal, NBI – nasal bias index, LSI – local synchrony index.

To assess the accuracy of our toolbox, we detected waves in retinal HD-MEA recordings taken at different ages and analyzed their spatiotemporal properties, which are very well described in the literature (Figure 2A). We binned the recordings into 5 age groups – 0 weeks (postnatal day or P0-P2), 0.5 weeks (P3-P5), 1 week (P6-P8), 1.5 weeks (P9-P11), and 2 weeks (P12-P14). We first assessed the occurrence of waves at the various age groups, both in terms of frequency (Figure 2B, **left**), which calculates the total occurrence of waves across the whole retina, or inter-wave-intervals (IWIs) (Figure 2B, **right**), which calculates the occurrence of waves per unit space of the retina. Both analyses reveal that waves are much more frequent in the 2^nd^ postnatal week than the 1^st^. We next assessed the areas of waves and found that they gradually become larger throughout development before shrinking back in late stage III (Figure 2C). When assessing the duration of waves, we observed that waves exhibit a gradual increase peaking at 1 week, before gradually decreasing in the 2^nd^ week (Figure 2D). Lastly, analyzing speed revealed waves were much faster in the 2^nd^ postnatal week than the 1^st^ (Figure 2E). Overall, these results are all consistent with previous reports (Figure 2A).

We next tested whether waves exhibit any location-specific biases during the first two postnatal weeks, both regarding initiation and propagation bias. We first assessed the initiation location of retinal waves at all of our age groups. Retinas from 0.5–1-week-old mice exhibited a slight initiation bias in temporal retina (Figure 2F), consistent with *in vivo* reports^16^. We next assessed the propagation direction of retinal waves at all of our age groups. Retinas from 1.5-week-old mice exhibited a markedly temporal-to-nasal propagation bias (Figure 2G–H), consistent with both *ex vivo*^15,22^ and *in vivo*^14^ reports. Lastly, we assessed local synchrony during retinal waves, a measure of how much activity is contained to the wave-front versus just ahead of the wave-front, and is a feature of retinal waves thought to be important for the refinement of RGC axons^19^. We found that local synchrony was strongest early during development and gradually became worse over time (Figure 2I).

### Coupling our toolbox with long HD-MEA recordings reveals the dynamic and stable nature of spatiotemporal properties of retinal waves.

**Figure 3:**
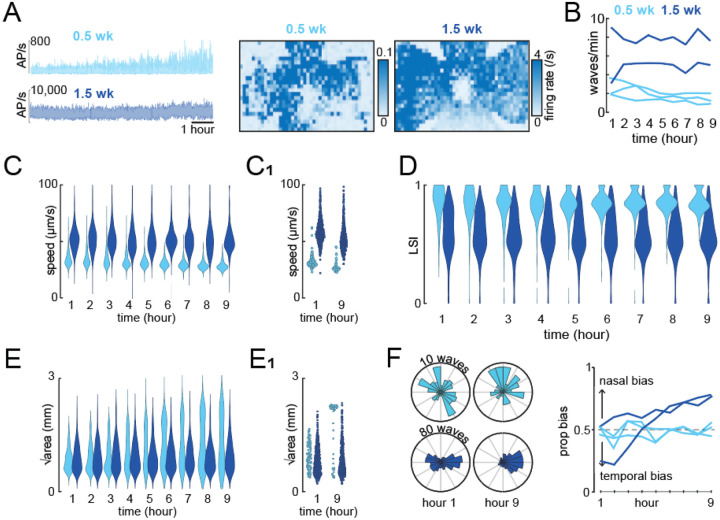
Long term recordings coupled with toolbox reveal the dynamic and stable structure of spatiotemporal properties of waves. A) Left: Overall retinal activity obtained from two nine-hour long HD-MEA recordings, one from a 0.5 week old mouse (light blue) and one from a 1.5 week old mouse (dark blue). Right: Heatmaps of mean firing rate of neurons recorded over the course of nine hours. N = 3039 waves across 3 mice for 0.5 week-old, N = 6892 waves across 2 mice for 1.5 week-old. B) Wave frequency across the 0.5 and 1.5 week old retina over the course of the nine hours. Each line represents data from one mouse. C) Wave speeds of all retinal waves recorded across all 0.5- and 1.5-week-old mice, binned per hour. C1) Wave speeds of retinal waves from a representative 0.5- and 1.5-week-old mouse at the first hour and ninth hour of the recording. D) Local synchrony computed for all retinal waves recorded across all 0.5- and 1.5-week-old mice, binned per hour. E) Wave size (calculated as $\sqrt{\text{Area}}$) of all retinal waves recorded across all 0.5- and 1.5-week-old mice, binned per hour. E1) Wave size of retinal waves from a representative 0.5- and 1.5-week-old mouse at the first hour and ninth hour of the recording. F) Left: polar histograms depicting wave direction in the first and ninth hour for a representative 0.5- and 1.5-week-old mouse. Right: Propagation bias calculated per hour over the course of the nine hours. Each line represents data from one mouse.

Having validated our toolbox with accurately revealing the known spatiotemporal properties of retinal waves during development, we next aimed to use the toolbox to make novel discoveries about retinal waves. One outstanding question about retinal waves is whether the spatiotemporal properties are stable or dynamic over the course of several hours. To begin to answer this question, we performed nine-hour long HD-MEA recordings of retinal waves in 0.5-week-old mouse (P5; n=3 mice; 3039 waves) and in 1.5-week-old mouse (P12; n = 2 mice; 6892 waves) (Figure 3A, **left**). All of our recording sampled across large areas of the retina and thus, combined with the nine-hour recording, enabled a high throughput assessment of the spatiotemporal properties of waves in individual mice (Figure 3A, **right**).

We found several spatiotemporal properties that were consistently stable across the duration of the nine-hour recording despite exhibiting age-dependent properties. For example, 0.5-week-old retinas exhibited less frequent waves than 1.5-week-old retinas, but both had stable frequencies over the nine-hour recordings (Figure 3B, **right**). Likewise, although wave speeds were slower for 0.5-week-old retinas than 1.5-week-old retinas, all wave speeds were stable throughout the duration of the recordings (Figure 3C and C1). Similarly, although local synchrony was higher for 0.5-week-old retinas than 1.5-week-old retinas, all local synchrony measures were stable throughout the duration of the recordings (Figure 3D).

Surprisingly, we found a couple spatiotemporal properties that were dynamic for some ages but stable for others. First, in all three of our recordings for 0.5 week-old-retinas, we observed that the distribution of waves areas was unimodal in the first hour but bimodal by the ninth hour, with both very large and very small waves present (Figure 3E and E1). In both of our recordings for 1.5 week-old-retinas, the distribution of wave areas is stable across the nine hours. Second, in both of our recordings for 1.5 week-old-retinas, the propagation of retinal waves did not initially exhibit a nasal-dominant bias but developed a strong nasal-dominant bias by the end of the ninth hour (Figure 3F). In all three of our recordings for 0.5 week-old-retinas, retinal waves exhibited stable symmetric propagation (no bias) across the nine hours. All together, these results demonstrate that the spatiotemporal properties of retinal waves exhibit distinct dynamic features depending on the age of the animal.

### The toolbox enables detection of retinal waves even in high noise environments.

A key method to discover circuits and factors that govern retinal waves is to use pharmacology or genetic manipulations that perturb retinal waves. Unless the manipulation completely abolishes retinal waves, determining the effect of the manipulation on the spatiotemporal properties of retinal waves is difficult. Calculations that are agnostic to retinal waves, like the STTC calculations^23^, allow information about how a manipulation affects distance-dependent synchrony, but not how other spatiotemporal properties of waves, like area or propagation bias, are affected. Thus, we tested whether our toolbox could continue to reliably detect retinal waves even under the most severe cases of neural activity manipulation. To do so, we utilized mice where the excitatory Gq-DREADD is expressed in all RGCs (using vglut2-cre) and performed 10-minute HD-MEA recordings before and after activating DREADDs with the DREADD ligand, clozapine-n-oxide (CNO). We furthermore performed these experiments in the 1^st^ and 2^nd^ postnatal week to test whether we could segregate cholinergic and glutamatergic waves in high noise conditions. Note that no control experiments to test for the presence of DREADDs or the ligand CNO are done since the point of this experiment was to artificially raise the floor of neural activity and test whether our toolbox could still segregate waves.

**Figure 4:**
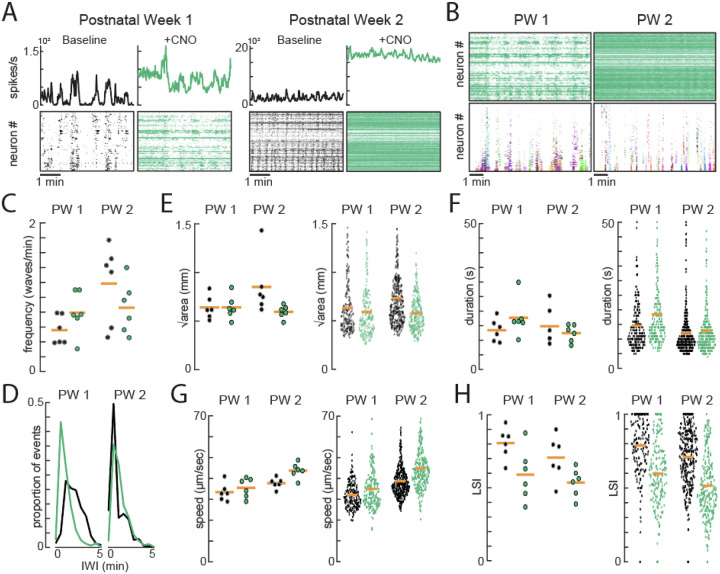
Using the toolbox to detect and analyze waves in recordings with very high noise. A) Raster plot of recorded spikes per neuron (bottom) and the sum of spikes across the retina (top) over time, both before (black) and after (green) application of CNO in vGlut2-cre/Gq-DREADD mice in the first postnatal week (left, P5) and the second postnatal week (right, P12). B) Raster plot of recorded spikes per neuron following the application of CNO in vGlut2-cre/Gq-DREADD mice in the first postnatal week (top left, P5) and the second postnatal week (top right, P12) and the detected waves in each recording (bottom left, P5 and bottom right, P12). Each color represents a unique wave ID (P5, N=40 waves; P12, N=42 waves). C) Frequency per mouse before (black) and after (green) application of CNO during the first postnatal week (PW1, N=6 mice) and second postnatal week (PW2, N=6 mice). D) Inter-wave-intervals (IWIs) between consecutive waves before (black) and after (green) application of CNO during PW1 and PW2, shown as a proportion of events at any given IWI. E-H) Area, duration, speed, and local synchrony (LSI) of individual waves (right) and the average per mouse (left) before (black) and after (green) application of CNO during PW1 (before CNO, N=191 waves across 6 mice; after CNO, N=206 waves across 6 mice) and PW2 (before CNO, N=372 waves across 6 mice; after CNO, N=286 waves across 6 mice).

For both the first and second postnatal week, CNO applied on Gq-DREADD-expressing retinas elicited a significant increase in the tonic firing rate of retinal neurons (Figure 4A), thereby increasing the overall “noise” in the HD-MEA recording. Our toolbox still successfully segregated retinal waves despite this significant increase in noise (Figure 4B) and found comparable number of waves before and after application of CNO (1^st^ week: n = 6 mice, before CNO = 191 waves, after CNO = 206 waves; 2^nd^ week: n = 6 mice, before CNO = 372 waves, after CNO = 286 waves) we assessed their spatiotemporal properties (Figure 4C–H). Most notably, following activation of Gq-DREADDs, retinal waves propagated faster and exhibited a strong decrease in local synchrony.

## Discussion

Here we report on a new tool that we built to segregate individual retinal waves and analyze their spatiotemporal properties. Our toolbox was initially designed to work on both MEA and calcium imaging data, but works with any dataset that has spatial and temporal information. We validated our toolbox on the known spatiotemporal properties of retinal waves during development: our toolbox correctly catches that the increasing frequency and speed of waves that occurs in the second week, and correctly identifies the propagation bias in the middle of the second postnatal week. In addition to calculating known spatiotemporal properties, we added novel analyses that have become relevant given recent publications^19^. We used this toolbox to analyze the spatiotemporal properties of retinal waves in ultra long recordings and demonstrated that spatiotemporal properties exhibit stable or dynamic nature in an age-dependent manner. We also demonstrated that our toolbox is capable of detecting and analyzing retinal waves even following the addition of pharmacological agents, enabling the assessment of how retinal waves are impacted by various manipulations. Lastly, our toolbox can be applied to non-retinal wave data, enabling the detection of events that are contiguous in space and time to study their spatiotemporal properties. Our toolbox can be used by running a pipeline of codes that are available on our Github page or by running a standalone graphical user interface (GUI) that we developed. In summary, our toolbox has broad applicability, has been extensively validated with known results from the literature, and is designed to be user friendly.

### Waves in the first postnatal week exhibit better properties for setting up retinotopic maps

A recent study demonstrated the Hebbian mechanism via which the actual propagation of retinal waves is critical for the refinement of retinal axons in the superior colliculus^24^. A key factor of retinal waves that is alluded from that work is that as a wave is propagating, neurons just outside of the retinal wave are generally not active, thus allowing for Hebbian plasticity mechanisms to refine retinal axons. We added a functionality to our toolbox to test the local synchrony of neural activity at the wavefront by tabulating the amount of activity at the wavefront compared to activity just outside of the wavefront. This analysis allowed us to determine that local synchrony at the wavefront is much higher in the first postnatal week than the second postnatal week, which is consistent with the fact that the bulk of retinotopic refinement occurs in the first postnatal week. Analysis of local synchrony will be useful for studies that aim to test how various environmental factors or neurodevelopmental disorders affect the key spatiotemporal properties of waves that are important for setting up retinotopic maps.

### Spatiotemporal properties of waves can be dynamic on an hourly basis

Although the spatiotemporal properties of retinal waves have been extensively studied during development^13,14^, little is known about how waves change throughout the day on an hourly basis. A possible limitation that has led to this lack of information is in the difficulty of segregating waves and analyzing their spatiotemporal properties. Long-term recordings are data-intensive, often consisting of thousands of retinal waves, and cannot efficiently be analyzed without some automation.

Our toolbox resolves this limitation and has allowed us to make a novel discovery regarding waves: Certain spatiotemporal properties of retinal waves can be dynamic or stable in an age-dependent manner. In our recordings, we noticed that wave frequency and wave speed were very stable at their expected age-dependent levels over the course of several hours: For example, wave frequency in the first postnatal week is low compared to the second postnatal week, but these frequencies remained stable over hours for both age groups. By contrast, wave areas exhibited stable properties in the second postnatal week but not the first, with wave areas initially exhibiting a unimodal distribution in the first hour of the recording and exhibiting a bimodal distribution after nine hours.

The dynamic nature of the spatiotemporal properties of waves could perhaps be explained by *ex-vivo* preparations dying over the course of nine hours, but there are several reasons why this explanation is unlikely to be true. First, within the same recording, whereas some spatiotemporal properties can be dynamic (area), others (frequency and speed) are very stable over nine hours, and one would expect the frequency of waves to change drastically if the tissue was dying. Second, the dynamic nature of wave areas is only true in the first postnatal week, just like how the dynamic nature of wave propagation is only true in the second postnatal week. Again, if tissue necrosis was the mechanism of the dynamic nature of the spatiotemporal property, we would expect this to similarly affect retinal waves in the first and second postnatal week. Together, these facts argue against tissue necrosis as an explanation of the dynamic nature of waves.

What then, are possible mechanisms that could cause a spatiotemporal property, like wave area, to be dynamic over the course of hours? One possibility is circadian modulation of retinal activity. Retinal neurons have their own clock proteins^26,28^ and it is possible that excitability of RGCs changes throughout the day, which could lead to broader coverage of the retina at different time periods. Another possible mechanism is that the retina is developing, even in an *ex-vivo* preparation. Future experiments will test which of these mechanisms, if not both, are involved in causing some spatiotemporal properties to be dynamic at specific ages.

The fact that waves are dynamic over the course of hours carries several important implications. One implication, related to methodology and research design, is that future work studying the impact of factors on the spatiotemporal properties of waves needs to be aware that some of these properties can be dynamic throughout the day. The propagation bias of retinal waves has garnered high interest recently^14,22^, and much work is currently being done to determine the exact circuit mechanisms that dictate this bias or whether mouse models of neurodevelopmental disorders exhibit an abnormal propagation bias. Another implication is that since the spatiotemporal properties of waves are critical for the development of the visual system, it now becomes critical to determine the mechanisms that set their dynamic nature. It is possible that certain brain states set the spatiotemporal properties of waves and thus could be critical for the healthy development of the visual system.

### Broad applicability of the toolbox – pharmacology and other datasets

Since the spatiotemporal properties of retinal waves are critical for instructing the development of the visual system, there is a lot of interest in identifying factors that perturb the spatiotemporal properties of waves. Unfortunately, tools and methods to study the spatiotemporal properties of waves are specifically designed to work under baseline conditions and fail once a manipulation is done to perturb retinal activity. Our DREADD experiments aimed to demonstrate that our ability to segregate and analyze retinal waves is maintained even under severe changes in the firing rate of retinal neurons. Thus, our toolbox enables high-throughput assessment of how retinal waves are perturbed by different pharmacological agents (external factors) or in mouse models of neurodevelopmental disorders. Importantly, the dataset generated from this study or future uses of our toolbox could be used to train machine learning algorithms to more rapidly detect retinal waves.

## Methods

### Ethics.

All animal procedures were approved by the Vanderbilt Animal Care and Use Program and conformed to the NIH *Guide for the Care and Use of Laboratory Animals*, the Public Health Service Policy, and the SFN Policy on the Use of Animals in Neuroscience Research.

### Animals.

All mice in this study were aged between 0–14 days and of both sexes and were housed in a 12/hr day/night cycle vivarium. Experiments studying the spatiotemporal properties of retinal waves over development as well as long recordings were done using C57BL/6J mice (Jackson stock: 000664). Experiments on assessing the spatiotemporal properties of waves following activity manipulations were done using litters from Vglut2-ires-cre::GqDREADD mice (Jackson stocks: 016963 and 026220).

### Method Details

#### Retina preparation.

Mice were euthanized using an overdose of isoflurane inhalation. Eyes were immediately enucleated and retinas were dissected in ice cold oxygenated under room lights (95% O_2_/ 5% CO_2_) Artificial CerebroSpinal Fluid (ACSF; in mM, 119 NaCl, 2.5 KCl, 1.3 MgCl_2_, 1 K_2_HPO_4_, 26.2 NaHCO_3_, 11 D-glucose, and 2.5 CaCl_2_). We chose to perform all experiments under room light since constant light has no effect on retinal waves across development^30^. Isolated retinas were mounted whole on a MaxWell Biosystems MaxOne chip. The chip was loaded onto a Maxwell Biosystems MaxOne Single-Well High-Density Multi-Electrode Array (HD-MEA) and the retina was secured using a MaxOne tissue holder. A perfusion system was used to perfuse the retina at a constant rate (2 mL/min) with oxygenated (95% O_2_/ 5% CO_2_) ACSF and maintained at a temperature of 32–34 °C. For all experiments, the ACSF is never recycled.

#### High-Density Multi-Electrode Array (HD-MEA) recordings.

A MaxWell Biosystems MaxOne Recording Unit Hub was connected to the HD-MEA and recorded the retinal activity detected by the electrodes on the MaxOne chip. For all recordings, the electrode configuration consisted of 1024 electrodes (12 reference electrodes, 1012 recording electrodes) distanced 87.5μm apart (MaxLab Live Scope predefined configuration Sparse1x). Following a 1-hour acclimation period, retinal activity was recorded. Only the action potentials were recorded (MaxLab Live Scope setting ‘Record Spikes Only’).

#### Ultra long recordings.

9-hour HD-MEA recordings of retinal activity were obtained using the same setup and configurations described above. Due to software limitations, three 3-hour recordings of the same retina were collected consecutively and concatenated post-experiment.

#### Manipulation of retinal waves.

To increase overall retinal activity, retinas of VGLUT2Cre::GqDREADD +/+ mice were dissected and loaded onto the HD-MEA. Following a 1-hour acclimation period, a 10-minute recording of retinal activity was collected, referred to as “baseline”. The DREADD agonist, Clozapine-N-oxide (CNO) was then bath-applied, followed by a 5-minute waiting period, at which point another 10-minute recording was collected, referred to as “+CNO”.

### Data Analysis Details.

#### Wave detection pipeline.

Hierarchal data (.h5) was uploaded into the wave detection script (written using MATLAB 2024b), outputting data organized in a way where every action potential has a time stamp and recorded channel. Combined with information about the spatial location of each channel, we reconstructed movies of retinal activity over time (x by y by t). **At this step, HD-MEA data matches the format of calcium imaging movies (x by y by t), so either types of data can be used moving forward.** To help isolate waves, we first filtered out the tonic firing of each by conducting a burst detection analysis. This algorithm dynamically detects baselines throughout the recording, allowing it to analyze the bursting patterns of neurons and distinguish between tonic and phasic activity. With only phasic activity remaining, the data was fed into the wave detection algorithm. This algorithm scans for active channels, or pixels, in the xyt movie. Once an active pixel is found, the algorithm determines whether this pixel has active neighboring pixels. If no, the algorithm discards that pixel and scans for the next active pixel. If yes, the algorithm collects spatial and temporal information about all pixels that are connected in space (xy) and time (t), forming a putative wave. Once all connected pixels in space and time are detected, the putative wave is given a unique ID tag, and the participating spatial and temporal pixels are given this ID tag in a resulting wave data matrix. The algorithm then seeks out the next active pixel and process repeats until all pixels in space and time have been scanned. When the algorithm is done processing the data, the output is a wave data matrix that is the same size as the raw data matrix (xyt).

#### Wave analysis pipeline.

The wave data matrices were uploaded into the wave analysis pipeline (written using MATLAB 2024b) for analysis of their spatiotemporal properties. This pipeline runs through each recording and analyzes the spatiotemporal properties of each wave in the current recording, compiling the results into a table. For a given recording, we calculated the following spatiotemporal frequencies:

Wave frequency was calculated as:

$$\text{wave}\;\text{frequency}=\left(\left(\overset{\text{numUniquePixels}}{\underset{\text{pixelID}=1}{\sum }}\frac{\text{numUniqueWaves}}{\text{recordingTime}}\right)/\text{numUniquePixels}\right)60$$

Where *pixelID* is the unique pixel number, *numUniquePixels* is the total number of unique pixels, *numUniqueWaves* is the number of unique waves that pass through a pixel, and *recordingTime* is the length of the recording in seconds. The calculation is converted from seconds to minutes, yielding waves/min.

Inter-wave intervals (IWIs) are computed in a similar manner as wave frequency, except IWIs are the minutes between each wave.

Wave area was calculated by isolating the wave into its own matrix, summating the wave in the third dimension (*xyt* to *xy*), counting the number of active pixels, and converting that value to μm^2^ (1 pixel = 87.5 μm).

Wave duration was calculated as:

$$\text{wave}\;\text{duration}=\left|t_{end}-t_{\text{start}}\right|$$

Where *t*_*end*_ is the last frame of activity for a unique wave and *t*_*start*_ is the first frame of activity for a unique wave.

Wave speed was calculated by using a Euclidean distance transform to measure the distances between the leading edge of the wave between pairs of 1-s time frames. The mean distance traveled by the leading edge was divided by the time between frames (1s) to get the speed of the wavefront in pixels/s, which was then converted to μm/s (1 pixel = 87.5 μm). Our speed measurements may differ from previous literature because we specifically chose to not calculate the speed of the wave using the center of mass of the wave at each frame since this does not adequately capture the speed of a radially propagating wave.

Wave initiation sites were calculated by finding the center of mass of the first frame of each wave. Wave initiation bias was calculated as:

$$IBI=\frac{\text{numN}}{\text{numN}+\text{numT}}$$

Where *IBI* is the initiation bias index, *numN i*s the number of waves that initiated on the nasal half of the retina, and *numT* is the number of waves that initiated on the temporal half of the retina. An *IBI* value closer to 1 indicates a nasal initiation bias, and a value closer to 0 indicates a temporal initiation bias. Initiation bias is assessed by halves of the retina (nasal and temporal initiation), rather than assessing in quadrants (nasal, temporal, dorsal, and ventral), because all four leaflets of the retina do not possess equal electrode coverage due to the MEA chip’s rectangular shape.

Wave vector flow field was calculated as:

For each wave, the flow of the wave among all participating pixels was calculated for the entire duration of the wave. To calculate the flow of the wave at each pixel, we generated a 3×3 grid of pixels centered on the pixel of interest and calculated the center of mass of the wave within that grid for the entire duration of the wave. The Euclidean distance of each center of mass for that pixel was then tabulated

$${\vec{v}}_{pix}=\overset{t_{end}}{\underset{t_{\text{start}}}{\sum }}v_{x}\hat{l}+v_{y}\hat{J}$$

Where, for a given wave, ${\vec{v}}_{pix}$ is the vector sum over time of a unique participating pixel, *t*_*start*_ is the first frame of the wave, and *t*_*end*_ is the last frame of the wave. $v_{x}\hat{l}$ and $v_{y}\hat{J}$ are the *x* and *y* vector magnitudes of the difference in the pixel’s grid center of mass between consecutive frames (1s).

The propagation direction of the wave is the average vector of all vectors in the flow field. Wave propagation bias was calculated as:

$$NBI=\frac{\text{numN}}{\text{numN}+\text{numT}+\text{numV}+\text{numD}}$$

Where *NBI* is the nasal bias index, *numN* is the number of waves that propagate in the nasal direction, *numT* is the number of waves that propagate in the temporal direction, numV is the number of waves that propagate in the ventral direction, and *numD* is the number of waves that propagate in the dorsal direction. A *NBI* value closer to 1 indicates more of a nasal propagation bias, while a value closer to 0 indicates less of a nasal propagation bias. A value of 0.25 indicates no propagation bias.

Local synchrony index (LSI) was calculated as:

$$LSI=\frac{\text{activit}y_{wf}}{\left(\text{activit}y_{wf}+\text{activit}y_{\text{aheadwf}}\right)}$$

For each wave and frames that are included in the duration of the wave, we tabulated both the amount of activity that is present at the leading wavefront (*activity*_*wf*_) and the amount of activity that is present ahead of the wavefront (*activity*_*aheadwf*_). For a given frame *F*_*t*_ and a subsequent frame *F*_*t+1*_, we subtracted *F*_*t+1*_ from *F*_*t*_ to obtain the activity ahead of the wavefront.


$$\text{activit}y_{\text{aheadwf}}=F_{t+1}-F$$

## Data Availability

Processed data will soon be available on our lab’s Github page: https://github.com/TiriacLabCodeShare/waveMiner. The non-processed dataset will also be uploaded onto that same Github page.

## Code Availability.

Analysis code will soon be available on our lab’s Github page: https://github.com/TiriacLabCodeShare/waveMiner.
